# Prevalence and Risk Factors of Depression between Patients with Parkinson’s Disease and Their Caregivers: A One-Year Prospective Study

**DOI:** 10.3390/healthcare10071305

**Published:** 2022-07-14

**Authors:** Yu Lee, Yung-Yee Chang, Ying-Fa Chen, Tsu-Kung Lin, Chi-Fa Hung, Yu-Jie Chiou, Liang-Jen Wang

**Affiliations:** 1 Department of Psychiatry, Kaohsiung Chang Gung Memorial Hospital and Chang Gung University College of Medicine, Kaohsiung 83301, Taiwan; lyu722@cgmh.org.tw (Y.L.); chifa@cgmh.org.tw (C.-F.H.); yujie6666@gmail.com (Y.-J.C.); 2 Department of Neurology, Kaohsiung Chang Gung Memorial Hospital and Chang Gung University College of Medicine, Kaohsiung 83301, Taiwan; changyy7@gmail.com (Y.-Y.C.); alpha0716@gmail.com (Y.-F.C.); tklin@cgmh.org.tw (T.-K.L.); 3 Department of Child and Adolescent Psychiatry, Kaohsiung Chang Gung Memorial Hospital and Chang Gung University College of Medicine, Kaohsiung 83301, Taiwan

**Keywords:** depression, prevalence, risk factors, Parkinson’s disease, caregiver

## Abstract

Depression is a common comorbidity in patients with Parkinson’s disease (PD) and in their caregivers. This study aimed to compare the prevalence and risk factors of depression between patients with PD and their caregivers. In total, 113 patients with PD and 101 caregivers were enrolled. Patients with PD were assessed using the Mini International Neuropsychiatric Interview, Unified Parkinson’s Disease Rating Scale (UPDRS), Activities of Daily Living (ADL), Hospital Anxiety and Depression Scale, Beck Hopelessness Scale, Brief Fatigue Inventory, Connor–Davidson Resilience Scale, and Big Five Inventory-10. Caregivers of patients with PD were also assessed using the above-mentioned instruments, with the exception of the UPDRS and ADL. During a 12-month follow-up period, depressive disorders were the most common psychiatric diagnosis of PD patients (27.4%) and their caregivers (17.8%). Depressive disorders were more prevalent in PD patients than in caregivers of PD patients throughout the entire follow-up phase. The severity of fatigue and severity of suicide risk were significantly associated with depression among patients with PD. The severity of pain and severity of anxiety were predictors of depression in caregivers of PD patients. The findings in this study provide references for early detection and treatment of depressive disorders in PD patients and their caregivers.

## 1. Introduction

Parkinson’s disease (PD) is a progressive, neurodegenerative disease that affects millions of people worldwide [1]. It is characterized by motor symptoms and non-motor symptoms. Among the non-motor symptoms, depression is the most incapacitating, affecting the PD patient’s quality of life, mortality, and even suicide risk [2,3]. Depressive disorders include major depressive disorder (MDD), persistent depressive disorder, other specified depressive disorder, premenstrual dysphoric disorder, and disruptive mood dysregulation disorder based on the DSM-V classification [4]. MDD, a common comorbidity in PD patients, is characterized by depressed mood, decreased interest, poor appetite, insomnia, cognitive impairment, pessimistic thinking, or even suicidal ideas/attempts [5,6]. The prevalence of the depressive disorder, ranging from 2.7% to 90%, among individuals with PD has varied widely in different studies [7]. It is recognized that clinically significant depression occurs in an estimated 40–50% of PD patients [8]. In general, a higher rate of depression is assessed when patient self-rated questionnaires are used than when structured interviews are conducted by experienced psychiatrists [9]. Only a few prior studies have used the structured clinical interview to report the prevalence of depression among patients with PD, which has ranged from 25.5% to 31.4% [10,11].

Previous studies have shown that the risk factors for depression in patients with PD include: a history of anxiety and/or depression before PD diagnosis, a low educational level and a family history of depression, severity of motor symptoms, disease stage, disease duration, daily levodopa equivalents dose, and the presence of non-motor symptoms such as sleep disturbance, anxiety, and hallucinations [12,13,14,15]. However, these studies have been restricted to a cross-sectional design and have yielded inconsistent results.

Previous studies on caregivers of patients with PD were focused on care burden or quality of life [16,17]; other studies mentioned that the depression rate among caregivers of patients with PD ranged from 14% to 35% [17,18]. All of these studies used self-administered questionnaires to detect depression rather than evaluations by a psychiatric specialist. One study from Taiwan used a structured clinical interview to diagnose the caregivers of PD patients and found that the morbidity of depressive disorder was 11.1% among caregivers, which is lower than that of prior studies using self-rated questionnaires [19]. 

Understanding the risk factors of depression may be helpful in developing strategies for preventing depression among caregivers of PD patients. There are a few studies on the associated factors of depression among caregivers of PD patients. They found that duration of PD, older age, low income, being a wife/female spouse, and hours spent caregiving are associated with depression among caregivers of PD patients [20,21,22]. Our prior study focused on the associated factors of depression in PD patients’ caregivers and found that duration of caregiving, severity of anxiety, and severity of fatigue were three significant associated factors for the development of depression [19]. The above-mentioned studies were cross-sectional, and the results were not consistent. Further follow-up study to investigate this important issue is warranted. 

There are scant follow-up studies that compare the prevalence and risk factors of depression in PD patients and their caregivers. In order to efficiently manage PD patients and their caregivers’ mental health, the aims of this follow-up study were to compare the prevalence and risk factors of depressive disorder between patients with PD and their caregivers.

## 2. Materials and Methods

### 2.1. Participants

All procedures performed in studies involving human participants were in accordance with the Declaration of Helsinki (1964) and its later amendments or comparable ethical standards. The Institutional Review Board at Chang Gung Memorial Hospital has approved this study (IRB No. 201702186B0).

This study used a prospective design with consecutive sampling. PD patients and their caregivers were recruited from the neurology ward or neurology outpatient clinic at a general hospital (Kaohsiung Chang Gung Memorial Hospital) from August 2018 to July 2020. This hospital has 2754 beds and provides services to 350 people with PD per year in southern Taiwan. Inclusion criteria were as follows: (1) individuals who were diagnosed with PD by an expert neurologist, (2) individuals who are the PD patient’s principal caregiver (“principal caregiver” is defined as “living with the patient and taking care of his/her daily needs”), and (3) individuals with the ability to understand the study procedure and who could provide written informed consent. Exclusion criteria: (1) individuals with a diagnosis of delirium or atypical parkinsonism (e.g., dementia with Lewy bodies, progressive supranuclear palsy, multiple system atrophy, corticobasal syndrome) or secondary parkinsonism; (2) individuals who were too weak to complete the questionnaire or clinical interview.

### 2.2. Study Procedure

Study procedures were as follows: (1) Newly-diagnosed patients visiting our collaborative care clinic or admitted to our ward were invited consecutively to take part in this study. Once our research assistant received a referral from the outpatient clinic or wards from in-charge doctors or case managers, our research assistant went to the above settings to contact the patients. After explaining the study procedure and aims, those who agreed to sign an informed consent form were enrolled in the study. (2) A senior psychiatrist (Dr. Y. Lee) made the psychiatric diagnoses using the Mini International Neuropsychiatric Interview (MINI) [23]. (3) Assessment tools, including the Brief Fatigue Inventory (BFI) [24] for measuring fatigue severity, Numeric Pain Rating Scale (NPRS) [25], Questionnaire Version of the List of Threatening Experiences (LTE-Q) [26] for social support and coping, Connor–Davidson Resilience Scale (CD-RISC) [27] for the ability to cope with stress, Hospital Anxiety and Depression Scale (HADS) [28] for the severity of depression and anxiety, Beck Hopelessness Scale (BHS) [29] for the severity of suicide risk, and the Big Five Inventory-10 (BFI-10) [30] for personality traits were used in the study. The BFI, NPRS, LTE-Q, CD-RISC, HADS, BHS, BFI-10, and clinical and demographic data were collected by a trained research assistant. (4) The above questionnaires and psychiatric diagnostic interviews were completed at baseline and at the 6-month and 12-month follow-up.

### 2.3. Statistical Analyses

Descriptive and inferential statistics were analyzed using SPSS for Windows V. 12.0. Kolmogorov–Smirnov test and Levene’s test were used to examine the normality and homoscedasticity of the sample, respectively (Table S1). Chi-Square and Mann–Whitney U tests were performed to test the differences in demographic data and clinical characteristics between subjects with and without a depressive disorder. We used Bonferroni correction to adjust for multiple testing in the correlation matrix. Logistic regression was used to test the factors associated with depressive disorder. Depression was set as a dependent variable, and patient and caregiver characteristics were set as independent variables. We calculated both the adjusted odds ratio (aOR) and the 95% confidence interval (CI). 

## 3. Results

A total of 113 PD patients and 101 caregivers finished our questionnaires during the 12-month follow-up period (Table S1). Table 1 demonstrates the demographic and clinical characteristics of the patients with Parkinson’s disease at a 12-month follow-up. Among the 113 patients, 67.3% (*n* = 76) were males. The average age of the patients was 65.4 ± 8.5 years. Their mean educational level was 11.0 ± 4.6 years, 90.3% were married, and 80.5% were currently unemployed. Their mean duration of illness was 8.5 ± 5.5 years. Sixty-five percent of the patients had comorbidity of one or more physical illnesses.

Of the 101 caregivers that successfully completed the 12-month follow-up study, 69.3% (*n* = 101) were females. The average age of the caregivers was 61.4 ± 10.7 years. Their mean educational level was 11.2 ± 4.6 years, 90.1% were married, and 71.3% were currently unemployed. Their mean duration of caring was 8.1 ± 5.3 years. Fifty-four percent of the caregivers had comorbidity of one or more physical illnesses (Table 2).

**Figure 1:**
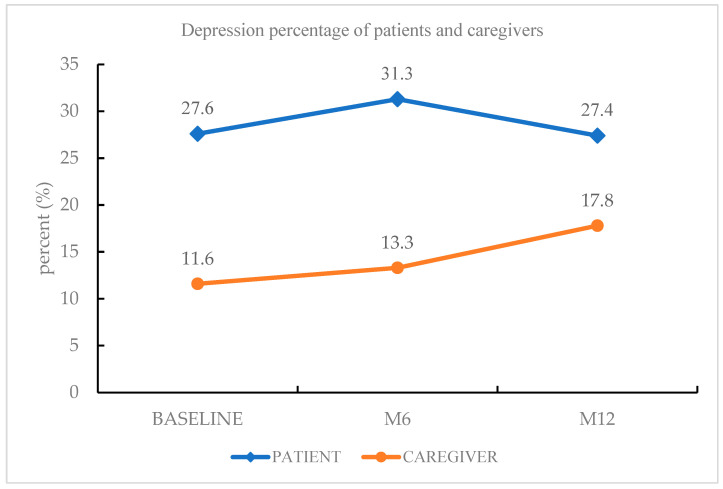
Depression morbidity of PD patients and caregivers.

The most common psychiatric diagnosis of PD patients during the follow-up period was depressive disorders (27.4%), followed by insomnia disorder (10.6%), rapid eye movement sleep behavior disorder (8.8%), and generalized anxiety disorder (7.1%). Among the depressive disorders, other specified depressive disorder (16.8%) was the most frequent diagnosis, followed by MDD (10.6%). Forty-four percent of patients had a psychiatric diagnosis. The most common psychiatric diagnosis of the caregivers was depressive disorders (17.8%), followed by insomnia disorder (14.9%) and generalized anxiety disorder (4.0%). Depressive disorders were more prevalent among PD patients than among caregivers of PD patients at baseline, at the 6-month follow-up phase, and at the 12-month follow-up phase (Figure 1).

In comparing depressed PD with non-depressed PD patients, depressed PD patients more often had higher BHS scores (10.29 ± 3.88 vs. 3.73 ± 2.87, *p* < 0.001), higher FSS scores (50.55 ± 12.34 vs. 23.11 ± 12.71, *p* < 0.001), lower CDRISC scores (19.16 ± 7.52 vs. 32.51 ± 8.07, *p* < 0.001), higher neuroticism scores (7.29 ± 1.77 vs. 5.61 ± 1.75, *p* < 0.001), and higher HADS-A scores (8.65 ± 4.55 vs. 3.23 ± 2.60, *p* < 0.001) than non-depressive PD patients (Table 1). When the above significant factors were analyzed relative to depressive disorders of PD patients at the 12-month follow-up using the stepwise forward model of logistic regression, severity of fatigue (odds ratio (OR) =1.09; 95% CI, 1.03–1.16; *p* < 0.05) and severity of suicide risk (OR = 1.43; 95% CI, 1.15–1.79; *p* < 0.05) were two significant risk factors (Table 3).

**Figure 2:**
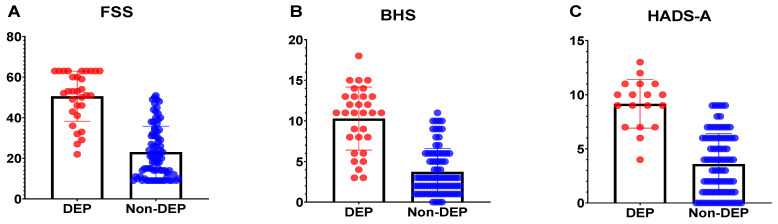
The psychometrics between patients or caregivers with depression and without depression.

Depressed caregivers more often had higher BHS scores (7.61 ± 4.59 vs. 2.60 ± 2.56, *p* < 0.001), higher FSS scores (32.39 ± 12.94 vs. 18.90 ± 8.70, *p* < 0.001), lower CDRISC scores (24.06 ± 8.08 vs. 32.23 ± 7.20, *p* < 0.001), and higher HADS-A scores (9.17 ± 2.26 vs. 3.60 ± 2.82 *p* < 0.001) than non-depressive caregivers (Table 2). When the above significant factors were analyzed relative to the depressive disorders of caregivers at the 12-month follow-up using the stepwise forward model of logistic regression, the severity of anxiety (OR = 1.73; 95% CI, 1.26–2.82; *p* < 0.05) was a significant risk factor (Table 4). Figure 2 demonstrated the scores distribution of FSS (Figure 2A) and BHS (Figure 2B) between Parkinson’s disease patients with depression and without depression. The scores distribution of the HADS-A (Figure 2C) between caregivers of Parkinson’s disease patients with depression and without depression.

The scores distribution of the Fatigue Severity Scale (Figure 2A, FSS) and Beck Helplessness Scale (Figure 2B, BHS) between Parkinson’s disease patients with depression (DEP) and without depression (Non-DEP). The scores distribution of the Hospital Anxiety and Depression Scale (Figure 2C, HADS-A) between caregivers of Parkinson’s disease patients with depression (DEP) and without depression (Non-DEP). 

Patients with PD were more often males (x^2^ = 28.52, *p* < 0.001) and had higher BHS scores (5.53 ± 4.32 vs. 3.50 ± 3.58, *p* < 0.001), higher FSS scores (30.64 ± 17.57 vs. 21.31 ± 10.83, *p* < 0.001), lower extraversion scores (4.59 ± 2.59 vs. 5.84 ± 2.45, *p* < 0.001), and higher neuroticism scores (6.07 ± 1.90 vs. 5.13 ± 1.65, *p* < 0.001) than their caregivers (Table S2). 

We excluded PD patients and caregivers who have past psychiatric history, family psychiatric history, and family suicide history and totally had 93 patients and 83 caregivers. After statistical analysis by logistic regression, associated factors of depressive disorders among PD patients and their caregivers were the same as original patients (*n* = 113) and caregivers (*n* = 101) (Tables S3–S6).

## 4. Discussion

There were limited previous studies that detected the prevalence and risk factors of depression between PD patients and their caregivers [31,32]. This prospective study reported the prevalence and risk factors of depressive disorder between patients with PD and their caregivers during a one-year follow-up. In our study, depressive disorder (27.4% and 17.8%) was the most common psychiatric diagnosis among both PD patients and their caregivers, respectively. Insomnia disorder (10.6% and 14.9%) was the second most frequent psychiatric diagnosis among PD patients and their caregivers, respectively. We found that depressive disorders were more prevalent in PD patients than in caregivers of PD patients at every follow-up phase. During the 12-month follow-up, the depression morbidity of PD patients showed a trajectory pattern, whereas the depression morbidity of PD patients’ caregivers showed a steadily increasing pattern (Figure 1). There were more elderly patients, more patients with higher pain severity, higher fatigue severity, and personality traits with less extraversion and more neuroticism among PD patients than their caregivers. These clinical characteristics may partially explain why depressive disorder was more prevalent among PD patients than among their caregivers. Of note, nearly 30% of the PD patients had clinical depression persistently for one year, and depression morbidity increased among caregivers of PD patients. Therefore, it is crucial to manage the depression of PD patients and their caregivers rigorously to improve their quality of life.

Depressive disorder was the most common psychiatric diagnosis in PD patients. This result is in line with that of previous studies, in which PD patients were commonly comorbid with depression, within a range of 40–50% [8]. A study from the USA examined 137 PD patients using the Structured Clinical Interview for DSMIV-TR Axis I Disorders, Research Version, Non-Patient edition (SCID) [33], and found that 43 (31.4%) of them were diagnosed as having depressive disorder [10]. In our study, 12 (10.6%) of the patients were diagnosed as having MDD, and 19 (16.8%) had depressive disorder not otherwise specified. These two studies suggest that patients with PD are quite often comorbid with depressive disorder. In addition, depression morbidity in both studies was within the range of 27.4–31.4%, which was lower than that of some studies of PD patients, which reported depression morbidity up to 50% [8]. The possible explanation is that both studies used a structured diagnostic interview by a clinician, which would render a lower morbidity rate than the self-rated questionnaires used in other clinical studies. Notwithstanding the lower morbidity rate of depression found when the clinician uses a structured diagnostic interview, the MINI or SCID, which are structured diagnostic interview instruments, are the gold standard for diagnosis, even beyond the psychiatric interview. 

In our study, depressive disorder was the most frequent psychiatric diagnosis among caregivers of PD patients, and the frequency increased steadily from baseline to the 12-month follow-up (from 11.6% to 13.3% and to 17.8%). This result is supported by previous studies, in which depression morbidity among caregivers of PD patients was within the range of 14–35% [17,18]. There are two clinical implications of this result: (1) our psychiatric diagnosis was reached using a structured clinical interview, which is more precise than the self-rated questionnaires used in previous studies; (2) this prospective study found that there was an increasing tendency toward depression among caregivers, and this should be given more attention by clinicians. 

After the baseline interview, our research assistant would notify PD patients’ in-charge neurologist if patients have a depressive disorder. Neurologist usually prescribed antidepressants to patients or transferred depressive PD patients to the psychiatric outpatient clinic by clinical judgment. Once depressive PD patients received medication treatment, their depression improved markedly. Whereas caregivers, after baseline interview, our research assistant would suggest caregivers refer to the psychiatric outpatient clinic; however, most caregivers refused to visit our clinic due to mental stigma, which is commonly seen in Taiwan. 

The risk factors for depression in PD patients were severity of fatigue and severity of suicide risk, and in caregivers, the risk factor was the severity of anxiety. The possible explanations for the discrepancies in risk factors between PD patients and their caregivers are: (1) PD patients had higher depression morbidity and disease-related weakness than their caregivers, which contributed to the risk factors of severity of suicide risk and severity of fatigue; (2) caregivers of PD patients had more of a care burden physically and mentally than did PD patients, which contributed to the risk factor severity of anxiety [34]. To our knowledge, this is the first study to compare the risk factors of depression between PD patients and their caregivers. More prospective studies should be conducted to confirm these findings.

The present study revealed two significant risk factors for depressive disorder among our PD patients: severity of fatigue (OR = 1.09; 95% CI, 1.03–1.16; *p* < 0.05), and severity of suicide risk (OR = 1.43; 95% CI, 1.15–1.79; *p* < 0.05). Suicide risk was the most robust risk of depression in PD patients in our study. O’Brien et al. (1987) investigated 98 patients with self-harm and found that a high severity of suicide ideation in patients was associated with severity of depression and with having depressive disorder [35]. This notion supports our result that suicide risk is one of the risk factors for depression among PD patients. Our prior cross-sectional study found that severity of anxiety (OR = 1.35; 95% CI, 1.18–1.55; *p* < 0.001), severity of suicide risk (OR = 1.12; 95% CI, 1.02–1.23; *p* < 0.05), and anxiolytics/hypnotics use (OR = 2.79; 95% CI, 1.23–6.31; *p* < 0.05) were three significant associated factors of depression in PD patients [36]. After a one-year follow-up, suicide risk increased and was the only persistent risk for depression. This finding suggests that suicide risk is a true risk contributing to depression in PD patients. Moreover, risk factors might change over time: as we found in this study, fatigue has become a new risk for depression in our patients. A possible explanation for this is that our PD patients experienced worse fatigue after the progression of the disease, and this physical exhaustion contributed to the development of depression [37]. 

This study found one significant risk factor for depressive disorder among our caregivers of PD patients with severity of anxiety (OR = 1.73; 95% CI, 1.26–2.82; *p* < 0.05). In this study, depressive caregivers had nearly two and a half times the severity of anxiety as non-depressive caregivers. Many patients with MDD were comorbid with an anxiety disorder or suffered from subsyndromal anxiety symptoms [38]. This comorbidity might support our finding that anxiety is an associated factor for depressive disorder. Possible explanations for why depressive caregivers more commonly have anxiety symptoms are that they have to deal with PD patients’ adverse effects of treatment, disease progression, and even a survival crisis, thus increasing the caregivers’ burden [39,40]. It is worth noting that our previous cross-sectional study found that duration of caregiving (OR = 1.28; 95% CI, 1.05–1.58), severity of anxiety (OR = 1.86; 95% CI, 1.3–2.53), and severity of fatigue (OR = 1.08; 95% CI, 1.01–1.16) were three significant associated factors of depression among caregivers of PD patients. One year later, the severity of anxiety increased, and this became a persistent risk for the development of depression among caregivers of PD patients.

If we excluded patients or caregivers with other psychiatric mental diseases, with a past psychiatric history, with family psychiatry history, or family suicide history, which factors might confound the authors cannot differ patients with PD or caregivers that already had previous and not PD-related reasons that could lead to depression, even doing this procedure, the results (Tables S2–S5) did not change significantly. We did not exclude anxiolytics/hypnotics use for Taiwan is the leading country in BZD prescription, where the prevalence of BZD use increased from 3.0% to 7.3% from 1997 to 2004 [41]. 

The follow-up study design to identify possible risk factors and the use of a structured clinical interview by psychiatrists are two strengths of this study. However, several limitations should be mentioned: (1) Our study design involved consecutive sampling, which may have led to sampling bias. (2) Our samples were from a general hospital, and may not be representative of the general population. (3) There were limited sample numbers of both patients and their caregivers, which limited the statistical power of the findings. Larger-scale studies of PD patients and their caregivers should be performed in the future to overcome this limitation. (4) In this study, we did not manage depression among PD patients and their caregivers. Consequently, we cannot understand the results of their treatment of depression.

## 5. Conclusions

To conclude, we found that depression was more prevalent in PD patients than in their caregivers. Furthermore, the risk factors for depressive disorder in PD patients were severity of fatigue and severity of suicide risk, and those for their caregivers were severity of pain and severity of anxiety. These findings give us new insight into the importance of conducting intervention programs directed toward ameliorating these risk factors to prevent these two groups from developing depression.

## Figures and Tables

**Table 1:** Demographic and clinical characteristics of the patients with Parkinson’s disease at 12-month follow-up (N = 113).

	Patients					
	Depressive N (%), N = 31	Non-Depressive N (%), N = 82	Total N (%), N = 113	z/χ^2^	Cohen’s D/Phi	*p*
**Gender (%)**				1.64	0.12	0.20
Male	18 (58.1)	58 (70.7)	76 (67.3)			
Female	13 (41.9)	24 (29.6)	37 (33.0)			
**Age, years mean (s.d.)**	64.39 ± 8.47	65.79 ± 8.53	65.41 ± 8.50	−0.64	−0.16	0.52
**Age of onset**	55.90 ± 9.29	57.38 ± 10.95	56.97 ± 10.50	−1.01	−0.15	0.31
**Duration of PD**	8.45 ± 5.54	8.48 ± 5.49	8.47 ± 5.48	−0.12	−0.005	0.91
**Years of education**	11.71 ± 4.64	10.66 ± 4.63	10.95 ± 4.63	−1.34	0.23	0.18
**Education**				0.25	0.05	0.62
Less than high school (<12)	12 (38.7)	36 (43.9)	48 (42.5)			
More than college (≥12)	19 (61.3)	46 (56.1)	65 (57.5)			
**Marital status**				0.00	0.001	0.99
Unmarried	3 (9.7)	8 (9.8)	11 (9.7)			
Married	28 (90.3)	74 (90.2)	102 (90.3)			
**Unemployment**	24 (77.4)	67 (81.7)	91 (80.5)	0.26	0.05	0.61
**Comorbid with other diseases**	18 (58.1)	55 (67.1)	73 (64.6)	0.80	−0.08	0.37
**Past psychiatric history**				3.87	0.19	0.049
No psychiatric history	23 (74.2)	73 (89.0)	95 (84.8)			
Depressive disorder	7 (22.6)	3 (3.7)	10 (8.8)			
Anxiety disorder	1 (3.2)	3 (3.7)	4 (3.5)			
Insomnia	2 (6.5)	3 (3.7)	5 (4.4)			
**Family psychiatric history**				3.04	0.03	0.39
No psychiatric history	29 (93.5)	78 (95.1)	107 (94.7)			
Depressive disorder	1 (3.2)	3 (3.7)	4 (3.5)			
Anxiety disorder	1 (3.2)	1 (1.2)	2 (1.8)			
**Family suicide history**	0	4 (4.9)	4 (3.5)	1.57	−0.12	0.21
**Anxiolytics/Hypnotics use**	8 (25.8)	24 (29.3)	32 (28.3)	0.13	−0.03	0.72
**NPRS (range)**	3.68 (0–10)	1.93 (0–8)	2.41 (0–10)	−2.73	0.63	0.006
**HADS total scores**	18.81 ± 6.61	8.32 ± 4.95	11.19 ± 7.18	−6.59	1.80	<0.001 *
HADS-D	10.16 ± 3.69	5.09 ± 3.17	6.48 ± 4.01	−5.96	1.47	<0.001 *
HADS-A	8.65 ± 4.55	3.23 ± 2.60	4.72 ± 4.04	−5.80	1.46	<0.001 *
**BHS**	10.29 ± 3.88	3.73 ± 2.87	5.53 ± 4.32	−6.68	1.92	<0.001 *
**FSS**	50.55 ± 12.34	23.11 ± 12.71	30.64 ± 17.57	−7.02	2.19	<0.001 *
**BFI-10**						
Extraversion	4.19 ± 2.23	4.74 ± 2.71	4.59 ± 2.59	−0.78	−0.22	0.44
Agreeableness	6.32 ± 1.47	7.00 ± 1.52	6.81 ± 1.53	−2.03	−0.45	0.042
Conscientiousness	7.97 ± 1.87	8.34 ± 1.96	8.24 ± 1.93	−1.19	−0.19	0.23
Neuroticism	7.29 ± 1.77	5.61 ± 1.75	6.07 ± 1.90	−4.12	0.95	<0.001 *
Openness	7.00 ± 1.34	6.80 ± 1.44	6.86 ± 1.41	−0.56	0.14	0.58
**LTEQ**	0.71 (0–3)	0.34 (0–4)	0.44 (0–4)	−1.94	0.42	0.052
**ADL scores**	88.55 ± 18.67	94.51 ± 15.55	92.88 ± 16.59	−2.27	−0.35	0.023
Highly dependent	2 (6.5)	4 (4.9)	6 (5.3)			
Moderate function	29 (93.5)	78 (95.1)	107 (94.7)			
**CDRISC**	19.16 ± 7.52	32.51 ± 8.07	28.85 ± 9.90	−6.26	−1.71	<0.001 *

NPRS—Numeric Pain Rating Scale; HADS—Hospital Anxiety and Depression Scale; HADS-D—Depression Scale; HADS-A—Anxiety Scale; BHS—The Beck Hopelessness Scale; FSS—Fatigue Severity Scale; LTEQ—Brief Life Event Questionnaire; CORISC—Connor–Davidson Resilience Scale; BFI-10—Big Five Inventory-10. Note: Bonferroni correction: *p* = 0.05/27 = 0.00185. Significant as * *p* < 0.00185.

**Table 2:** Demographic and clinical characteristics of the caregivers at 12-month follow-up (N = 101).

	Caregivers					
	Depressive N (%), N = 18	Non-Depressive N (%), N = 83	Total N (%), N = 101	z/χ^2^	Cohen’s D/Phi	*p*
**Gender (%)**				0.74	0.09	0.39
Male	4 (22.2)	27 (32.5)	31 (30.7)			
Female	14 (77.8)	56 (67.5)	70 (69.3)			
**Age, years mean (s.d.)**	60.44 ± 10.17	61.63 ± 10.83	61.42 ± 10.67	−0.67	−0.11	0.50
**Duration of caring**	7.89 ± 4.71	8.20±5.49	8.14 ± 5.34	−0.09	−0.06	0.93
**Years of education**	10.17 ± 4.58	11.43 ± 4.58	11.20 ± 4.58	−1.06	−0.28	0.29
**Education**				0.05	−0.02	0.83
Less than high school (<12)	7 (38.9)	30 (36.1)	37 (36.6)			
More than college (≥12)	11 (61.1)	53 (63.9)	64 (63.4)			
**Marital status**				0.46	0.07	0.50
Unmarried	1 (5.6)	9 (10.8)	10 (9.9)			
Married	17 (94.4)	74 (89.2)	91 (90.1)			
**Unemployment**	14 (77.8)	57 (69.9)	72 (71.3)	0.45	−0.07	0.50
**Comorbid with other diseases**	10 (55.6)	44 (53.0)	54 (53.5)	0.04	0.02	0.85
**Past psychiatric history**				7.85	0.28	0.005
No psychiatric history	13 (72.2)	78 (94.0)	91 (90.1)			
Depressive disorder	4 (22.2)	1 (1.2)	5 (5.0)			
Anxiety disorder	0	1 (1.2)	1 (1.0)			
Insomnia	4 (22.2)	2 (2.4)	6 (5.9)			
**Suicide history**	1 (5.6)	0	1 (1.0)	4.66	0.22	0.031
**Family psychiatric history**				0.23	−0.03	0.89
No psychiatric history	17 (94.4)	77 (94.4)	94 (93.1)			
Depressive disorder	1 (5.6)	5 (6.0)	6 (5.9)			
Anxiety disorder	0	1 (1.2)	1 (1.0)			
**Family suicide history**	0	5 (5.0)	5 (5.0)	1.14	−0.11	0.29
**Anxiolytics/Hypnotics use**	3 (16.7)	9 (10.8)	12 (11.9)	0.48	0.07	0.49
**NPRS (range)**	2.33 (0–7)	1.19 (0–10)	1.40 (0–10)	−2.38	0.52	0.017
**HADS total scores**	19.00 ± 4.97	7.46 ± 4.96	9.51 ± 6.64	−5.92	2.32	<0.001 *
HADS-D	9.83 ± 2.96	3.86 ± 2.88	4.92 ± 3.68	−5.65	2.04	<0.001 *
HADS-A	9.17 ± 2.26	3.60 ± 2.82	4.59 ± 3.46	−5.77	2.18	<0.001 *
**BHS**	7.61 ± 4.59	2.60 ± 2.56	3.50 ± 3.58	−4.42	1.35	<0.001 *
**FSS**	32.39 ± 12.94	18.90 ± 8.70	21.31 ± 10.83	−4.14	1.22	<0.001 *
**BFI-10**						
Extraversion	6.00 ± 2.70	5.81 ± 2.41	5.84 ± 2.45	−0.27	0.07	0.79
Agreeableness	6.17 ± 1.30	6.65 ± 1.56	6.56 ± 1.52	−1.34	−0.33	0.18
Conscientiousness	8.28 ± 1.36	8.16 ± 1.81	8.18 ± 1.73	−0.17	0.07	0.88
Neuroticism	6.17 ± 1.58	4.90 ± 1.58	5.13 ± 1.65	−2.54	0.80	0.011
Openness	6.39 ± 1.09	6.42 ± 1.42	6.42 ± 1.37	−0.04	−0.02	0.97
**LTEQ**	1.83 (0–3)	0.37 (0–15)	0.63 (0–15)	−2.58	0.57	0.01
**CDRISC**	24.06 ± 8.08	32.23 ± 7.20	30.77 ± 7.97	−3.78	−1.07	<0.001 *

NPRS—Numeric Pain Rating Scale; HADS—Hospital Anxiety and Depression Scale; HADS-D—Depression Scale; HADS-A—Anxiety Scale; BHS—The Beck Hopelessness Scale; FSS—Fatigue Severity Scale; LTEQ—Brief Life Event Questionnaire; CORISC—Connor–Davidson Resilience Scale; BFI-10—Big Five Inventory-10. Note: Bonferroni correction: *p* = 0.05/26 = 0.00192. Significant as * *p* < 0.00192.

**Table 3:** Associated factors of depressive disorder among patients at the 12-month follow-up: logistic regression analysis.

Item	β	S.E.	Wald	Odds Ratio	C.I.	*p*
**FSS**	0.09	0.03	7.98	1.09	1.03–1.16	0.005 *
**BHS**	0.36	0.11	9.95	1.43	1.15–1.79	0.002 *

FSS—Fatigue Severity Scale; BHS—The Beck Helplessness Scale: * *p* < 0.05.

**Table 4:** Associated factors of depressive disorder among caregivers at the 12-month follow-up: logistic regression analysis.

Item	β	S.E.	Wald	Odds Ratio	C.I.	*p*
**BHS**	0.23	0.14	2.68	1.25	0.96–1.64	0.101
**CDRISC**	0.04	0.06	0.39	1.04	0.92–1.17	0.532
**FSS**	0.07	0.04	2.55	1.07	0.99–1.16	0.111
**HADS-A**	0.55	0.25	4.75	1.73	1.26–2.82	0.029 *

HADS-A—Hospital Anxiety and Depression Scale-Anxiety scale; CORISC—Connor–Davidson Resilience Scale; BHS—The Beck Helplessness Scale; FSS—Fatigue Severity Scale. * *p* < 0.05.

## Data Availability

The data of the current study are available from the corresponding author on reasonable request.

## Supplementary Materials

The following supporting information can be downloaded at: https://www.mdpi.com/article/10.3390/healthcare10071305/s1, Table S1: The test of normality and homoscedasticity of the main outcomes of the sample; Table S2: Demographic and clinical characteristics of the patients with Parkinson’s disease and their caregivers at 12-month follow-up (N = 214). Table S3: Demographic and clinical characteristics of the patients with Parkinson’s disease at 12-month follow-up (N = 93); Table S4: Demographic and clinical characteristics of the caregivers at 12-month follow-up (N = 83); Table S5: Associated factors of depressive disorder among patients at the 12-month follow-up: logistic regression analysis; Table S6: Associated factors of depressive disorder among caregivers at the 12-month follow-up: logistic regression analysis.
