# Supplementary material for: Prevalence and Risk Factors of Depression between Patients with Parkinson’s Disease and Their Caregivers: A One-Year Prospective Study
Source: Healthcare (Basel). 2022 Jul 14;10(7):1305. doi: 10.3390/healthcare10071305 (PMC9318994; doi:10.3390/healthcare10071305)
Supplement: Supplementary file 1 [file healthcare-10-01305-s001.zip › healthcare-1773317-supplementary.pdf]

Supplementary Materials

Table S1. The test of normality and homoscedasticity of the main outcomes of the sample.

	Kolmogorov–Smirnov test		Levene's test	
	Statistics	p-value	Statistics	p-value
**Patients**				
HADS	0.13	<0.001	3.80	<0.001
HADS-D	0.15	<0.001	2.29	0.13
HADS-A	0.15	<0.001	22.84	<0.001
BHS	0.19	<0.001	4.92	0.03
FSS	0.11	0.001	0.34	0.56
CDRISC	0.18	<0.001	4.43	0.04
LTEQ	0.41	<0.001	8.72	0.004
**Caregivers**				
HADS	0.09	0.031	0.03	0.85
HADS-D	0.11	0.008	0.18	0.67
HADS-A	0.13	<0.001	4.21	0.04
BHS	0.24	<0.001	14.04	<0.001
FSS	0.14	<0.001	5.80	0.02
CDRISC	0.12	0.001	0.34	0.56
LTEQ	0.36	<0.001	19.20	<0.001

HADS—Hospital Anxiety and Depression Scale; HADS-D—Depression Scale; HADS-A—Anxiety Scale; BHS—The Beck Hopelessness Scale; FSS—Fatigue Severity Scale; LTEQ—Brief Life Event Questionnaire; CDRISC—Connor–Davidson Resilience Scale; BFI-10—Big Five Inventory-10.

Table S2. Demographic and clinical characteristics of the patients with Parkinson's disease and their caregivers at 12-month follow-up (N = 214).

	Patients N (%), N=113	Caregivers N (%), N=101	Total N (%), N=214	z/χ²	Cohen's D/ Phi	*p*
**Gender (%)**				28.52	0.37	<0.001*
Male	76(67.3)	31(30.7)	107(50.0)			
Female	37(33.0)	70(69.3)	107(50.0)			
**Age, years mean (s.d.)**	65.41±8.50	61.42±10.67	63.52±9.77	-2.66	0.41	0.008
**Age of onset**	56.97±10.50					
**Duration of PD**	8.47±5.48					
**Duration of caring**		8.14±5.34				
**Years of education**	10.95±4.63	11.20±4.58	11.07±4.60	-0.37	-0.05	0.71
**Education**				0.76	0.06	0.38
Less than high school (<12)	48(42.5)	37(36.6)	85(39.7)			
More than college (≧12)	65(57.5)	64(63.4)	129(60.3)			
**Marital status**				0.002	-0.003	0.97
Unmarried	11(9.7)	10(9.9)	21(9.8)			
Married	102(90.3)	91(90.1)	193(90.2)			
**Unemployment**	91(80.5)	72(71.3)	163(76.2)	2.51	0.11	0.11
**Comorbid with other diseases**	73(64.6)	54(53.5)	127(59.3)	2.74	-0.11	0.10
**Past psychiatric history**				1.28	-0.08	0.26
No psychiatric history	96(85.0)	91(90.1)	187(97.4)			
Depressive disorder	10(8.8)	5(5.0)	15(7.0)			
Anxiety disorder	4(3.5)	1(1.0)	5(2.3)			
Insomnia	5(4.4)	6(5.9)	11(5.1)			
**Suicide history**	0	1(1.0)	1(0.5)	1.12	0.07	0.29
**Family psychiatric history**				1.57	0.03	0.67
No psychiatric history	107(94.7)	94(93.1)	201(93.9)			
Depressive disorder	4(3.5)	6(5.9)	10(4.7)			
Anxiety disorder	2(1.8)	1(1.0)	3(1.4)			
**Family suicide history**	4(3.5)	5(5.0)	9(4.2)	0.26	0.04	0.61
**Anxiolytics/Hypnotics use**	32(28.3)	12(11.9)	44(20.6)	0.13	-0.20	0.72
**NPRS (range)**	2.41(0-10)	1.40(0-10)	1.93(0-10)	-2.59	0.42	0.01
**HADS total scores**	11.19±7.18	9.51±6.64	10.40±6.96	-1.61	0.24	0.11
HADS-D	6.48±4.01	4.92±3.68	5.74±3.93	-2.81	0.41	0.005
HADS-A	4.72±4.04	4.59±3.46	4.66±3.77	-0.28	0.03	0.78
**BHS**	5.53±4.32	3.50±3.58	4.57±4.10	-3.83	0.51	<0.001*
**FSS**	30.64±17.57	21.31±10.83	26.23±15.47	-3.53	0.64	<0.001*
**BFI-10**						
Extraversion	4.59±2.59	5.84±2.45	5.18±2.60	-3.85	-0.50	<0.001*
Agreeableness	6.81±1.53	6.56±1.52	6.70±1.53	-1.34	0.16	0.18

Conscientiousness	8.24±1.93	8.18±1.73	8.21±1.84	-0.71	0.03	0.48
Neuroticism	6.07±1.90	5.13±1.65	5.63±1.84	-3.73	0.03	<0.001*
Openness	6.86±1.41	6.42±1.37	6.65±140	-2.26	0.53	0.024
**LTEQ**	0.44(0-4)	0.63(0-15)	0.53(0-15)	-0.15	-0.14	0.88
**ADL scores**	92.88±16.59					
High function	107(94.7)					
Moderate function	6(5.3)					
**CDRISC**	28.85±9.90	30.77±7.97	29.76±9.07	-1.23	-0.21	0.22

NPRS—Numeric Pain Rating Scale; HADS—Hospital Anxiety and Depression Scale; HADS-D—Depression Scale; HADS-A—Anxiety Scale; BHS—The Beck Hopelessness Scale; FSS—Fatigue Severity Scale; LTEQ—Brief Life Event Questionnaire; CORISC—Connor–Davidson Resilience Scale; BFI-10—Big Five Inventory-10. Note: Bonferroni correction: p=0.05/25=0.002. Significant as * p < 0.002.

Table S3. Demographic and clinical characteristics of the patients with Parkinson's disease at 12-month follow-up (N = 93).

	Patients					
	**Depressive N (%), N=22**	**Non-depressive N (%), N=71**	**Total N (%), N=93**	**z/χ²**	**Cohen's D/ Phi**	***p***
**Gender (%)**				1.91	0.14	0.17
Male	12(54.5)	50(70.4)	62(66.7)			
Female	10(45.5)	21(29.6)	31(33.3)			
**Age, years mean (s.d.)**	63.18±7.20	65.70±9.04	65.11±8.67	-1.38	-0.31	0.17
**Age of onset**	53.64±8.08	56.80±11.42	56.05±10.77	-1.70	-0.32	0.09
**Duration of PD**	9.50±5.98	8.95±5.54	9.08±5.62	-0.32	0.10	0.75
**Years of education**	10.73±4.97	10.76±4.58	10.75±4.65	-0.18	-0.01	0.86
**Education**				0.27	-0.05	0.60
Less than high school(<12)	11(50.0)	31(43.7)	42(45.2)			
More than college(≧12)	11(50.0)	40(56.3)	51(54.8)			
**Marital status**				0.52	-0.08	0.47
Unmarried	3(13.6)	6(8.5)	9(9.7)			
Married	19(86.4)	65(91.5)	84(90.3)			
**Unemployment**	18(81.8)	58(81.7)	76(81.7)	0.00	-0.001	0.99
**Comorbid with other diseases**	13(59.1)	48(67.6)	61(65.6)	0.54	-0.08	0.46
**Anxiolytics/Hypnotics use**	4(17.4)	19(26.8)	23(24.7)	0.66	-0.08	0.42
**NPRS (range)**	3.59(0-8)	2.01(0-8)	2.39(0-8)	-2.26	0.59	0.024
**HADS total scores**	18.82±6.71	8.41±4.96	10.87±6.98	-5.76	1.76	<0.001*
HADS-D	10.05±3.76	5.04±3.14	6.23±3.91	-5.23	1.44	<0.001*
HADS-A	8.77±4.66	3.37±2.66	4.65±3.96	-4.96	1.42	<0.001*
**BHS**	9.91±4.29	3.86±2.92	5.29±4.17	-5.38	1.65	<0.001*
**FSS**	49.45±12.05	23.92±12.55	29.96±16.49	-5.97	2.08	<0.001*
**BFI-10**						
Extraversion	4.36±2.40	4.86±2.83	4.74±2.73	-0.51	-0.19	0.61
Agreeableness	6.18±1.68	6.97±1.55	6.78±1.61	-1.85	-0.49	0.07
Conscientiousness	7.91±2.00	8.46±1.78	8.33±1.84	-1.20	-0.29	0.23
Neuroticism	7.18±1.74	5.56±1.76	5.95±1.88	-3.44	0.93	0.001*
Openness	6.68±1.29	6.86±1.46	6.82±1.41	-0.70	-0.13	0.49
**LTEQ**	0.82(0-3)	0.32(0-4)	0.44(0-4)	-2.11	0.55	0.035
**ADL scores**	87.95±19.86	94.14±12.71	93.44±14.91	-2.36	-0.37	0.018
Highly dependent	1(4.5)	3(4.2)	4(4.3)			
Moderate function	21(95.5)	68(95.8)	89(95.7)			
**CDRISC**	20.32±8.35	32.28±8.28	29.45±9.71	-4.91	-1.44	<0.001*

NPRS—Numeric Pain Rating Scale; HADS—Hospital Anxiety and Depression Scale; HADS-D—Depression Scale; HADS-A—Anxiety Scale; BHS—The Beck Hopelessness Scale; FSS—Fatigue Severity Scale; LTEQ—Brief Life Event Questionnaire; CORISC—Connor–Davidson Resilience Scale; BFI-10—Big Five Inventory-10. Note: Bonferroni correction: p= 0.05/24=0.0021. Significant as * p < 0.0021.

Table S4. Demographic and clinical characteristics of the caregivers at 12-month follow-up (N = 83).

	Caregivers					
	Depressive N (%), N=12	Non-depressive N (%), N=71	Total N (%), N=83	z/χ²	Cohen's D/ Phi	*p*
**Gender (%)**				0.02	0.01	0.90
Male	4(33.3)	25(35.2)	29(34.9)			
Female	8(14.8)	46(64.8)	54(65.1)			
**Age, years mean (s.d.)**	61.33±11.46	61.97±10.02	61.97±10.02	-0.07	-0.06	0.94
**Duration of caring**	7.92±4.17	8.29±5.67	8.29±5.67	-0.15	-0.07	0.88
**Years of education**	10.50±4.98	11.80±4.42	11.80±4.42	-0.74	-0.28	0.46
**Education**				0.004	-0.01	0.95
Less than high school (<12)	4(33.3)	23(32.4)	27(32.5)			
More than college (≧12)	8(66.7)	48(67.6)	56(67.5)			
**Marital status**				0.00	0.001	0.99
Unmarried	1(8.3)	6(8.5)	7(8.4)			
Married	11(91.7)	65(91.5)	76(91.6)			
**Unemployment**	8(66.7)	47(66.2)	55(66.3)	0.001	-0.003	0.98
**Comorbid with other diseases**	7(58.3)	36(50.7)	43(51.8)	0.24	0.05	0.63
**Anxiolytics/Hypnotics use**	1(8.3)	6(8.5)	7(8.4)	0.00	-0.001	0.99
**NPRS (range)**	2.08(0-7)	1.07(0-10)	1.07(0-10)	-1.64	0.44	0.10
**HADS total scores**	18.67±4.70	7.24±4.99	7.24±4.99	-4.97	2.36	<0.001*
HADS-D	9.50±3.03	3.82±2.94	3.82±2.94	-4.60	1.90	<0.001*
HADS-A	9.17±1.80	3.42±2.80	3.42±2.80	-5.00	2.44	<0.001*
**BHS**	7.83(1-16)	2.59(0-11)	3.35(0-16)	-3.76	1.39	<0.001*
**FSS**	33.42±12.60	18.77±8.86	20.89±10.73	-3.75	1.35	<0.001*
**BFI-10**						
Extraversion	6.25±2.63	5.80±2.32	5.87±2.36	-0.66	0.18	0.51
Agreeableness	6.17±1.40	6.70±1.60	6.63±1.58	-1.19	-0.35	0.23
Conscientiousness	8.50±1.45	8.04±1.84	8.11±1.79	-0.60	0.28	0.55
Neuroticism	6.00±1.54	4.93±1.58	5.08±1.61	-1.81	0.69	0.07
Openness	6.17±1.12	6.39±1.49	6.36±1.44	-0.60	-0.16	0.55
**LTEQ**	2.17(0-15)	0.31(0-3)	0.53(0-15)	-2.29	0.61	0.022
**CDRISC**	26.50±7.99	32.15±7.27	31.34±7.59	-2.40	-0.74	0.017

NPRS—Numeric Pain Rating Scale; HADS—Hospital Anxiety and Depression Scale; HADS-D—Depression Scale; HADS-A—Anxiety Scale; BHS—The Beck Hopelessness Scale; FSS—Fatigue Severity Scale; LTEQ—Brief Life Event Questionnaire; CORISC—Connor–Davidson Resilience Scale; BFI-10—Big Five Inventory-10. Note: Bonferroni correction: p= 0.05/22=0.00227. Significant as * $p < 0.00227$.

Table S5. Associated factors of depressive disorder among patients at the 12-month follow-up: logistic regression analysis.

Item	β	S.E.	Wald	Odds ratio	C.I.	*p*
**FSS**	0.12	0.03	12.88	1.13	1.06-1.21	<0.001*
**BHS**	0.35	0.12	8.43	1.41	1.12-1.78	0.004*

FSS—Fatigue Severity Scale; BHS—The Beck Helplessness Scale. * $p < 0.05$.

Table S6. Associated factors of depressive disorder among caregivers at the 12-month follow-up: logistic regression analysis.

Item	β	S.E.	Wald	Odds ratio	C.I.	*p*
**FSS**	0.03	0.05	0.30	1.03	0.94-1.12	0.58
**HADS-A**	0.90	0.29	9.52	2.47	1.39-4.38	0.002*

HADS-A—Hospital Anxiety and Depression Scale-Anxiety Scale; FSS—Fatigue Severity Scale. * $p < 0.05$.
